# Visualization of gaseous iodine adsorption on single zeolitic imidazolate framework-90 particles

**DOI:** 10.1038/s41467-021-24830-1

**Published:** 2021-07-23

**Authors:** Yuting Lei, Guihua Zhang, Qinglan Zhang, Ling Yu, Hua Li, Haili Yu, Yi He

**Affiliations:** 1 National Collaborative Innovation Center for Nuclear Waste and Environmental Safety, School of National Defence Science & Technology, Southwest University of Science and Technology, Mianyang, PR China; 2 SUSTech Core Research Facilities, Southern University of Science and Technology, Shenzhen, PR China

**Keywords:** Imaging studies, Reaction kinetics and dynamics

## Abstract

Zeolitic imidazolate frameworks (ZIFs) are very useful as high-capacity iodine (I_2_) adsorbents. The adsorption performance is usually probed by measuring a statistical average property over an entire sample consisting of a large number of ZIF particles, leaving the interparticle heterogeneity information among individuals. Here we report a dark-field microscopy (DFM) method to visualize gaseous I_2_ adsorption on single ZIF-90 particles in situ and in real time. The adsorption of I_2_ is found to alter the scattering spectrum of ZIF-90 particles, inducing a distinct color change from bluewhite to yellow. According to correlating the adsorption amount of gaseous I_2_ with the change of B value from DFM images, we quantitatively image the adsorption process and estimate the related kinetic parameters at the single particle level. Single particle measurements clarify the large particle-to-particle heterogeneity in adsorption reactivity and significant adsorption activity improvement of ZIF-90 after introduction of linker defects, which provides a microscopic understanding of the structure-activity relationship. We further demonstrate the capacity of this strategy for studying gaseous I_2_ adsorption on single ZIF-91 particle as a derivative of ZIF-90 to illustrate the generality.

## Abstract (web summary)

Zeolitic imidazolate frameworks are promising as high-capacity iodine adsorbents. Here the authors image the gaseous I_2_ adsorption on single ZIF-90 particles, clarifying the inter-particle heterogeneity in adsorption reactivity and performance improvement after introduction of linker defects .

## Introduction

The capture of gaseous iodine (I_2_) has been attracting intense interest^1,2^. The gaseous I_2_ with a high mobility is the volatile product of uranium fission and iodide oxidation by atmospheric ozone^3–5^. It can cause radiation damage, air contamination, climate change, and atmospheric particle formation^6–8^, which constitutes a serious threat to the ecological environment and public health. The serious harm of gaseous I_2_ requires developing high-performance porous adsorbents for capturing it. As a metal-organic framework (MOF) subfamily, zeolitic imidazolate framework (ZIF) based on imidazolate and transition metal ion is one such material that is capable of trapping gaseous I_2_ within the nanopore. The trapping capacity of ZIF for I_2_ is ascribed to the match in size and charge-transfer (CT) interaction between I_2_ molecule and aromatic imidazole ring^9–11^.

The majority of studies on the capture process of gaseous I_2_ by ZIF have relied on theoretical simulation, X-ray diffraction, and calorimetry. However, the structural heterogeneity among different ZIF particles in a sample is easily obscured by the aforementioned ensemble analysis methods. The properties and structures of individual particles are simply averaged in the ensemble sample consisting of billions of ZIF particles, which restricts the understanding of the structure-activity relationship. On the other hand, some attempts in the improvement of the capture of I_2_ confirm that it can amorphize ZIF-8 with distort pore apertures to kinetically trap I_2_^12^. Investigation on the dynamics of the reaction intermediate states is thus the key to clarify the kinetics, which is highly essential to design and develop active ZIF adsorbents. In order to obtain the kinetic, real-time monitoring the adsorption process of I_2_ at the single ZIF particle level is an important and challenging avenue in practice.

**Fig. 1:**
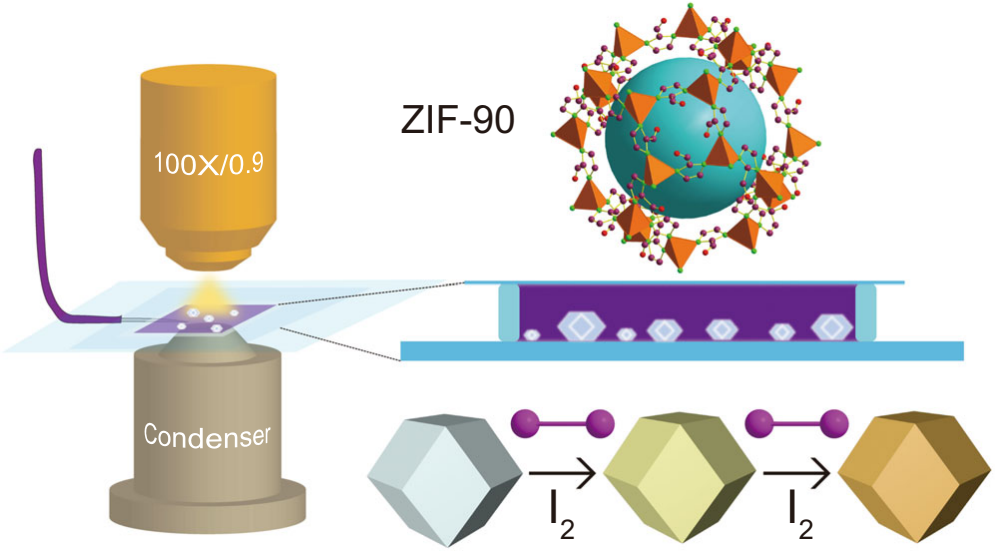
Schematic illustration of visualizing gaseous I_2_ adsorption on single ZIF-90 particles by dark-field microscopy. DFM setup, the structure of ZIF-90, microchamber, and the color variation of ZIF-90 particle before and after adsorption of gaseous iodine.

Here, we investigate the dynamic of the gaseous I_2_ adsorption on single ZIF-90 particles in situ and in real time with dark-field microscopy (DFM) (Fig. 1), a simple and powerful tool that can image individual nanoparticles with both high resolution and high contrast. Upon the introduction of gaseous I_2_, the B component of the DFM image of single ZIF-90 particles significantly decreases. This process accompanies distinct change in color and scattering peak, which enables and facilitates visual readout of the capture process of gaseous I_2_ by single ZIF-90 particle. By correlating the B value of the DMF image with adsorption amount of gaseous I_2_, we qualitatively determine the diffusion coefficient of gaseous I_2_ within ZIF-90 at the single-particle level. We observe the quite heterogeneous adsorption capacity of different individual ZIF-90 particles. Furthermore, we will show that the introduction of more defects will greatly enhance the adsorption rate and amount of gaseous I_2_ on single ZIF-90 particles. Meanwhile, this method is appropriate for studying other ZIF particles such as ZIF-91 as a derivative of ZIF-90. These findings offer single-particle visualization and mechanistic understanding of defect-promoted I_2_ adsorption in ZIF-90, thus proving the efficient avenue for revealing the structure-adsorption performance relationship.

## Results

### Visual observation of gaseous I_2_ adsorption on single ZIF-90 particles

**Fig. 2:**
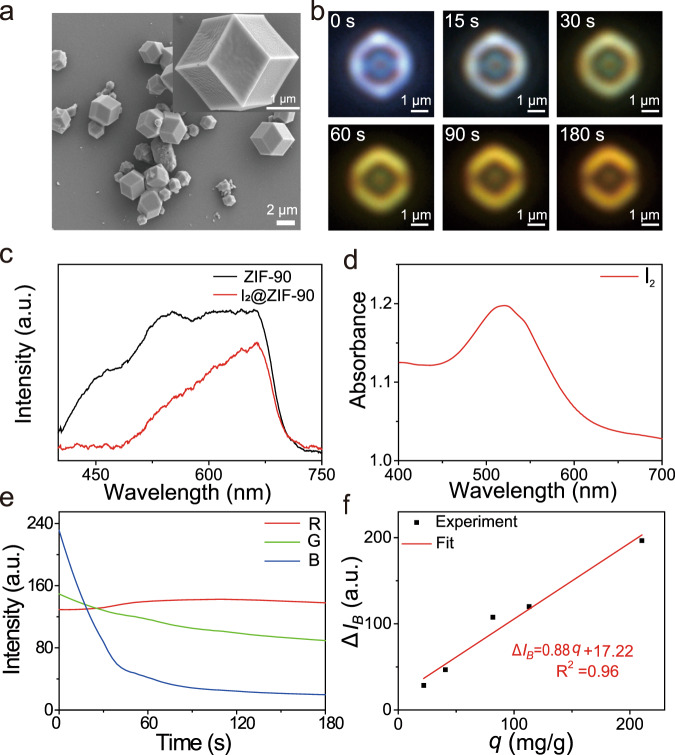
Structure characterization of ZIF-90 particles and in situ DFM data of gaseous I_2_ adsorption on single ZIF-90 particles. **a** SEM image of ZIF-90 particles. **b** Time-lapsed DFM images of gaseous I_2_ adsorption on single ZIF-90 particles (The concentration of gaseous I_2_ is 1 mM). **c** Scattering spectra of ZIF-90 particles before and after capturing I_2_. **d** The UV–vis adsorption spectrum of gaseous I_2_. **e** RGB values of DMF vs reaction time for capture of gaseous I_2_ by single ZIF-90 particle. **f** Plot of the change of B value (△*I*_*B*_) from the DFM image as a function of adsorption amount of I_2_ (*q*).

The ZIF-90 particles with a rhombic dodecahedron morphology and broad size distribution from 0.6 to 2.2 μm are synthesized by the previously reported protocol (Fig. 2a, Supplementary Figs. 1 and 2)^13^. Powder XRD data confirms that the obtained patterns match the simulated one for ZIF-90, demonstrating the good purity of the sample (Supplementary Fig. 3). In order to evaluate the porosity, the N_2_ adsorption isotherm is measured at 77 K (Supplementary Fig. 4). The Brunauer-Emmett-Teller (BET) area and pore size for ZIF-90 particles are calculated to be 897 m^2^·g^−1^ and 18.7 Å, which are close to the reported values^14^.

To image the gaseous I_2_ on single ZIF-90 particles, we dropcast the ZIF-90 dispersion on a microchamber, and the gaseous I_2_ is pumped. As shown in Fig. 2b, single ZIF-90 particle can be resolved by DFM, showing a nearly white color, with a touch of blue. This is because the scattering spectrum of ZIF-90 particles displays a broad peak covering the whole visible-light region (400–700 nm) (Fig. 2c), which is similar to that of polystyrene (PS) microspheres with diameter of 10 μm (the common whispering gallery-mode (WGM) resonators) (Supplementary Fig. 5)^15–17^. Recently, various MOFs have been confirmed to serve as WGM resonators as an alternative to PS microspheres^18–20^. Accordingly, the ZIF-90 particles are able to serve as WGM resonators for confining most of visible-light photons in their microstructures, resulting in a broad scattering peak (Supplementary Fig. 5a).

Fig. 2b shows some snapshots of the DFM imaging movie at different reaction time (Supplementary movie 1). As the adsorption process of gaseous I_2_ on single ZIF-90 particle proceeds, the color of the edge of the particle begins to yellow at 30 s. As the reaction time continues to increase, the entire ZIF-90 particle surface turns to yellow at 90 s. When the reaction time arrives at 180 s, the color of the ZIF-90 particle will no longer be altered, corresponding to the maximum capture capacity of single ZIF-90 particle for gaseous I_2_ (Fig. 2b). Correspondingly, the scattering spectrum of ZIF-90 particles after adsorption of I_2_ shows a significant intensity decrease in the blue light region with a wavelength around 400 to 525 nm (Fig. 2c), suggesting that the gaseous I_2_ can absorb the blue light, in line with the absorption spectrum of gaseous I_2_ (Fig. 2d). Accordingly, the ZIF-90 particle after capture of I_2_ exhibits a yellow color based on the complementary color principle (the complementary color to blue is yellow). Also, we perform molecular dynamics (MD) simulation to study the gas diffusion and optical responses of the I_2_-loaded ZIF-90 particle. The calculated results suggest that ZIF-90 includes two independent I sites and a maximum of 8 I_2_ molecules is accommodated inside each *β* cage (Supplementary Fig. 6a). The smaller *α* cage is too constrained to allow for loading I_2_. The I_2_ molecules are highly mobile within the *β* cage, but it is difficult that I_2_ molecules diffuse from one cage to another (Supplementary movie 2 and Supplementary Fig. 6b). Furthermore, the optical absorption properties of ZIF-90 with different I_2_ loading amounts are also calculated. As depicted in Supplementary Fig. 6c, pure ZIF-90 has no capacity for absorbing visible light, while the I_2_-loaded ZIF-90 can strongly absorb blue light (a wavelength between 400 and 525 nm). Meanwhile, the higher I_2_ loading amount, the stronger absorption for blue light. These results are in accord with the experimental data as shown in Fig. 2b, c.

The successful adsorption of I_2_ by ZIF-90 particles is further demonstrated by several characterizations. First, most of the XRD peaks remain at similar 2*θ* positions but are weaken, indicating that the diffusion process of gaseous I_2_ retains the host crystallinity (Supplementary Fig. 3). Second, the BET surface area of ZIF-90 particles dramatically decreases to 4.38 m^2^·g^−1^ after adsorption of I_2_ (Supplementary Fig. 4), mainly because of the filling of I_2_ molecules in the pores of ZIF-90. The elemental mapping analysis from the SEM image reveals the uniform distribution of I, C, N, O, and Zn elements in the surface of I_2_-loaded ZIF-90 particles (Supplementary Fig. 7). Besides, we employ the focused ion beam scanning electron microscopy (FIB-SEM) to investigate the internal adsorption of I_2_. Supplementary Fig. 8 shows the SEM images of I_2_-loaded ZIF-90 slices created by FIB under different adsorption time and the corresponding elemental mapping of the cross section for Zn and I. It can be seen that a very uniform elemental distribution of Zn and I is found in the cross section of I_2_-loaded ZIF-90 microparticle. These results indicate that the adsorbed I_2_ is evenly distributed in ZIF-90 particle. The corresponding I/Zn molar ratio increases with the increase of the adsorption time from 0 to 180 s and then levels off when the adsorption time exceeds 180 s (Supplementary Fig. 9), suggesting that the adsorption of gaseous I_2_ by ZIF-90 achieves an equilibrium state. Third, XPS studies are carried out to identify the chemical forms of I_2_. As depicted in Supplementary Fig. 10, the I 3*d* peaks are decomposed into double peaks of I_2_ (632.1 eV, 620.6 eV) and I_3_^−^ (630.4 eV, 619 eV)^21^. The production of I_3_^−^ proves the existence of the chemical adsorption and CT interaction between I_2_ and imidazole-2-carboxaldehyde (ICA)^22^. Additionally, the adsorbed I_2_ can be eluted from ZIF-90 particles with absolute EtOH, confirming that this adsorption process is reversible (Supplementary Fig. 11).

**Fig. 3:**
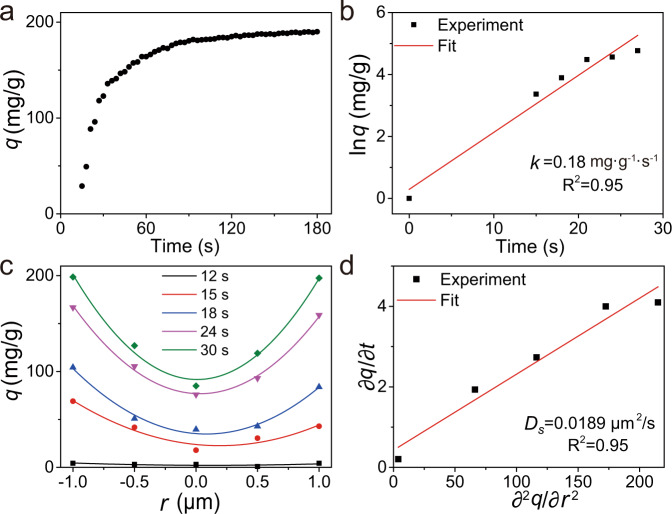
Kinetic analysis and determination of diffusion coefficient. **a** Adsorption kinetic of gaseous I_2_ onto a single ZIF-90 particle. **b** Pseudo-first order model for adsorption of gaseous I_2_ to calculate the apparent rate constant (*k*). **c** Spatial and temporal variability of I_2_ absorption amount distributions in line profiles. **d** Diffusion coefficient (*D*_*s*_) estimated from Fick’s second law.

In addition, the DFM images of single ZIF-90 particles can be deconstructed into RGB chrominance information. As the color of the ZIF-90 particle from bluewhite to yellow, the B value of the DFM image decreases successively (Fig. 2e). The corresponding X ray/synchrotron data is provided by using transmission electron microscopy-energy-dispersive X-ray spectroscopy (TEM-EDS). Both △*I*_*B*_ and I/Zn molar ratio follow a similar trend with increasing absorption time (Supplementary Fig. 12), revealing that the color change of the DFM image is induced by adsorption of gaseous I_2_ on ZIF-90 particles. We apply the variation of the B value (△*I*_*B*_) to quantitatively estimate the adsorption of gaseous I_2_ on ZIF-90 particles via correlating △*I*_*B*_ with adsorption amount of I_2_ (*q*) (Supplementary Fig. 13 and Fig. 2f). The relationship between △*I*_*B*_ and *q* can be fitted into a linear equation, △*I*_*B*_ = 0.88*q* + 17.22. Using this equation, we examine the adsorption kinetics of gaseous I_2_ on ZIF-90 particles. Fig. 3a shows the reaction time-dependence of *q* from single ZIF-90 particle. There is a good linear correlation between ln *q* and reaction time, obeying pseudo-first order model (Fig. 3b), and the apparent rate constant (*k*) is calculated to be 0.18 mg·g^−1^·s^−1^. Besides, under the assumption that the diffusivity coefficient (*D*_*s*_) is constant during the adsorption reaction, this process is capable of being described by Fick’s second law:

1 $$\frac{\partial C}{\partial t}={D}_{s}\frac{{\partial }^{2}C}{\partial {r}^{2}}$$

Where *t* and *r* are reaction time and the distance from the ZIF-90 particle center. By extracting spatial and temporal variability of gaseous I_2_ onto a single ZIF-90 particle in line profiles (Fig. 3c), the *D*_*s*_ is estimated to be 0.0189 μm^2^/s (Fig. 3d), which is comparable to that of other reported values^23^. Taken all together, this powerful DFM imaging approach can not only monitor the adsorption process of gaseous I_2_ on single ZIF-90 particle in real time and in situ, but also estimate the kinetic models and parameters as well as diffusion coefficients.

### Interparticle heterogeneity in adsorption reactivity

**Fig. 4:**
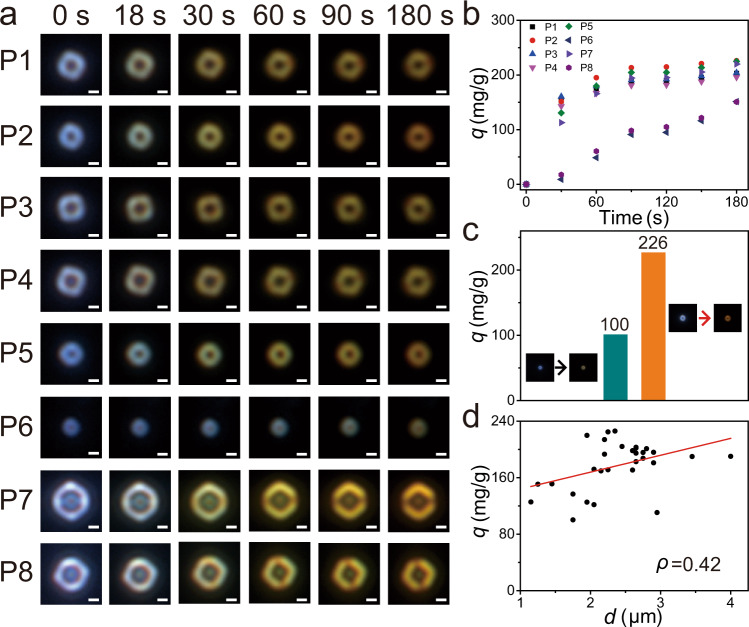
Imaging the gaseous I_2_ adsorption on ZIF-90 particles. **a** Time-resolved DFM images of gaseous I_2_ adsorption on eight representative ZIF-90 particles (scale bar: 1 μm) and (**b**) the corresponding adsorption kinetic. **c** Comparison of the absorption amount (*q*) between two ZIF-90 particles. The inset shows the DFM image of the ZIF-90 particles before and after adsorption of I_2_. **d** Correlation plot and the corresponding Pearson’s correlation coefficient (*ρ*) between absorption amount and diameter of ZIF-90 particles.

The present DFM method permits simultaneously imaging the adsorption reactivity of multiple ZIF-90 particles. Fig. 4a is a series of DFM images of gaseous I_2_ adsorption on eight representative ZIF-90 particle obtained in situ, labeled with P1 to P8. Although all the eight ZIF-90 particles gradually undergo a color change from bluewhite to yellow within 180 s, the change magnitudes and rates of the B value are distinct, indicating different adsorption amount and dynamic toward gaseous I_2_ for different ZIF-90 particles. For example, P1–P5, and P7 firstly approach to adsorption equilibrium at 90 s, followed by P6 and P8 (Fig. 4b). The adsorption amount of P2 for gaseous I_2_ is about 226 mg/g, which is much higher than that of P8 (~150 mg/g). Further statistical absorption amounts of 30 individual ZIF-90 particles reveal a significant particle-to-particle variation (Supplementary Fig. 14), and a 2.26-fold variation on the adsorption amounts of single ZIF-90 particles is found as shown in Fig. 4c. These data demonstrate the interparticle heterogeneity in the adsorption reactivity for gaseous I_2_. This level of information in the adsorption process is impossible to be achieved by using conventional spectral measurement on bulk sample.

It is generally assumed that the heterogeneity is related to the particle size. Taking P6 and P7 for example (Fig. 4b), P7 with a large size (3.8 μm) exhibits faster adsorption kinetic and more adsorption amount (*q* = 220 mg/g) than that of P6 with a small size (1.8 μm) (*q* = 151 mg/g). However, the size of P7 is very similar to P8 (3.6 μm), but the adsorption capacity of P7 is strikingly different from P8 (*q* = 150 mg/g) as illustrated in Fig. 4b. Therefore, the particle size of the ZIF-90 particles is not remarkably correlated with the adsorption amount for gaseous I_2_. This assumption is further supported via correlation analysis with a Pearson correlation coefficient of +0.42 (Fig. 4d), meaning that the particle size and adsorption amount have no strong relationship with each other. Previous fluorescence microscopy imaging studies infer that the heterogeneity of MOFs is attributed to the interior defect level caused by fluctuations in the preparation conditions^24,25^. The imperfect crystal structure of ZIF-90 particles are testified by SEM image (Fig. 2a). Some ZIF-90 particles have very regular rhombic dodecahedron shape, implying the good crystallinity, while others display serious crystal dislocation and rough surface (Fig. 2a). These results suggest that the heterogeneity of ZIF-90 particles in adsorption reactivity for gaseous I_2_ is dominated by particle-to-particle defect level differences.

### Defect-dominated adsorption performance of single ZIF-90 particles

**Fig. 5:**
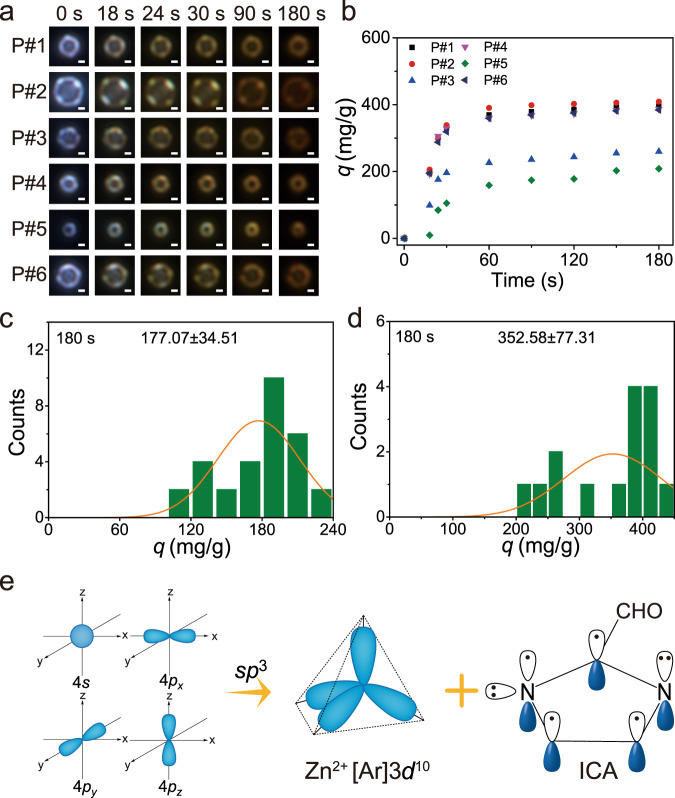
Visualization of gaseous I_2_ adsorption on defected ZIF-90 particles. **a** Time sequential DFM images of gaseous I_2_ adsorption on six representative defected ZIF-90 particles (scale bar: 1 μm) and (**b**) the corresponding adsorption kinetic. Histograms of the adsorption amount (*q*) for (**c**) ZIF-90 and (**d**) defected ZIF-90 particles. **e** Formation of *sp*^3^ orbitals from 4*s* and 4*p* orbitals of Zn^2+^ and molecular orbital structure of ICA.

In addition to investigating the interparticle heterogeneity, another noticeable feature of this imaging method is that it permits for evaluating the adsorption performance of defected ZIF-90 at the single-particle level. The inherent defect of ZIF-90 is relatively scarce, and water postsynthetic treatment is validated to be an efficient avenue for introducing more defects into ZIF-90 according to the generation of linker defects in the framework (Supplementary Fig. 15)^26^. The structure characterizations of the defected ZIF-90 before and after adsorption of gaseous I_2_ are depicted in Supplementary Figs. 16–19. Using the time sequential DFM images, we then quantify the kinetics of six representative defected ZIF-90 particles (Fig. 5), marked with P#1 to P#6. It is observed that the adsorption rate of defected ZIF-90 particles increases considerably. For instance, all the six defected ZIF-90 particles can attain adsorption chemical equilibrium within 60 s, which is apparently shorter than that of ZIF-90 particles (Figs. 5b and 4b). The statistical average value of the apparent rate constant for defected ZIF-90 particles is improved twice as much as that of the primary ZIF-90 particles (Supplementary Figs. 20 and 21). The same trends occur for the increase in the adsorption amount of I_2_ and diffusivity coefficient (Fig. 5c–d, and Supplementary Figs. 20–21). These data confirm that the defects in ZIF-90 particles play an important role in the adsorption of I_2_, which can increase adsorption rate, adsorption amount, and diffusivity coefficient.

In general, the CT interaction between electron-deficient I_2_ molecule and electron-rich aromatic ring of the imidazole is responsible for the capture of I_2_ by ZIF-90 particles (Supplementary Fig. 23)^9,11^. In the ZIF-90, the electron configuration of Zn^2+^ is [Ar]3*d*^10^, and the 4*s* and three 4*p* orbitals (*p*_*x*_, *p*_*y*_, and *p*_*z*_) hybridize to generate four vacant *sp*^3^ orbitals, which coordinate with the unpaired electron on the nitrogen atoms of the ICA to form Zn-N coordinate covalent bonds (Fig. 5e). This coordination interaction usually results in the delocalization of the electrons, decreasing the electron density of the imidazole ring. To depict the change of the electron density, we analyze the electrostatic potential (ESP) of ICA before and after coordination with Zn^2+^. The results, as depicted in Supplementary Fig. 24, clearly indicate that the Zn^2+^-ICA complex is more electron deficient as compared to ICA. However, when the linker defects are introduced, partial Zn-N bonds will be broken (Supplementary Fig. 15), which alleviates the delocalization of n electrons in N atoms from ICA, leading to the increase of the electron density. Owing to the high electron density of the defected ZIF-90, it favors the CT interaction between I_2_ and ICA aromatic ring, promoting the adsorption of I_2_ on ZIF-90 particles. Additionally, it also should be noted that we can not completely exclude the influence of the potential interference from grain boundary issues in the particles or purity of crystallinity on the adsorption of gaseous I_2_ due to the large particle-to-particle heterogeneity.

### Visual observation of gaseous I_2_ adsorption on single ZIF-91 particles

**Fig. 6:**
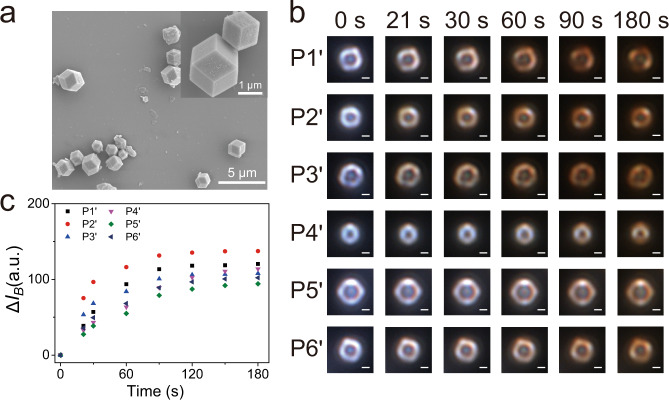
Visually monitoring gaseous I_2_ adsorption on individual ZIF-91 particles. **a** SEM image of ZIF-91 particles. **b** Time-resolved DFM images of gaseous I_2_ adsorption on six representative ZIF-91 particles (scale bar: 1 μm) and (**c**) the corresponding adsorption kinetic. △*I*_*B*_ represents the change of B value of the DFM images.

As an illustration of the generality of this imaging method for studying in situ the adsorption of I_2_, another ZIF material, ZIF-91 that is synthesized by reduction of ZIF-90 using NaBH_4_ is investigated as well (Supplementary Fig. 25). The XRD pattern, SEM image and elemental mapping demonstrate the successful preparation of ZIF-91 with the capacity of capturing I_2_ (Fig. 6a, Supplementary Figs. 26 and 27). Fig. 6b shows the time-resolved DFM images of gaseous I_2_ adsorption on six representative ZIF-91 particles, labeled with P1’ to P6’. Apart for P4’, the corners of other five ZIF-91 particles start to become yellow at ~ 21 s. After reaction for 90 s, the △*I*_*B*_ values of all the five ZIF-91 particles level off, corresponding to the adsorption equilibrium state (Fig. 6c). The large heterogeneity in the adsorption kinetic of individual particles is also observed as displayed in Fig. 6c and Supplementary Fig. 28. These observations are consist with the reaction trends obtained in the ZIF-90 particles. Overall, the present imaging strategy is extremely suitable to investigate the I_2_ adsorption by various ZIF materials.

## Discussion

In conclusion, we have demonstrated that the DFM method allows the visualization of gaseous I_2_ adsorption on single ZIF-90 particles. By correlating the △*I*_*B*_ with adsorption amount of I_2_, we can quantitatively determine the adsorption rate, adsorption amount, and diffusivity coefficient at the single particle level. DFM image analysis reveals the large heterogeneity in adsorption activity for I_2_ among different ZIF-90 particles because of the distinct particle-to-particle defect level. In addition, the present imaging method permits us to compare the adsorption performance of ZIF-90 particles before and after introduction of linker defects. The experimental results show that the linker defects result in the electron density increase of the imidazole ring, greatly promoting the adsorption of I_2_ on the defected ZIF-90 particles. Furthermore, the generality of this imaging method is also affirmed by showing the capacity for studying gaseous I_2_ adsorption on single ZIF-91 particles. This strategy using in situ DFM approach provides a fundamental understanding of the structure-performance relationship, which is potential to guide rational design and development of high-efficient porous MOF adsorbents.

## Methods

### Materials

Imidazole-2-carboxaldehyde, polyvinylpyrrolidone, 2-methylimidazole, and sodium borohydride are obtained from Aladdin Chemical Reagent Co., Ltd. Iodine is purchased from Sangon Co., Ltd. Zinc nitrate hexahydrate, tert-butyl alcohol, n-hexane, methanol, and ethanol are obtained from Chengdu Kelong Chemicals Co., Ltd.

### Preparation of ZIF-90 particles

The ZIF-90 particles are prepared by a simple one-pot method^13^. Briefly, 0.02 g polyvinylpyrrolidone and 0.12 g imidazole-2-carboxaldehyde are dissolved into 9 mL of deionized water, which is stirred for 3 min at 25 °C. After that, 10 mL of 0.033 mM zinc nitrate solution dissolved in tert-butyl alcohol is injected into the above solution under vigorous stirring for 10 min at 30 °C. The ZIF-90 particles are collected by centrifugation (3600 × *g* for 2 min), washed three times with methanol, and resuspended in absolute alcohol.

### Preparation of defected ZIF-90 particles induced by water treatment

For preparation of defected ZIF-90 particles^27^, 5 mg ZIF-90 is added to 5 mL deionized water. Subsequently, the solution is heated to 50 °C maintained at this temperature for 1 h, and the defected ZIF-90 particles are collected by centrifugation (3600 × *g* for 2 min) and dried at 70 °C for 12 h.

### Preparation of ZIF-91 particles

The ZIF-91 particles are synthesized by reduction of ZIF-90 by NaBH_4_^14^. ZIF-90 particles (0.1 g) are dispersed in 10 mL of 1.56 mM NaBH_4_ methanol solution and refluxed for 24 h. Then, the reaction solution is filtered. The resulting solid sample is washed twice with methanol, which is further exchanged with methanol for 1 day to obtain ZIF-91 particles. Then, the prepared ZIF-91 is dried at 70 °C for 12 h.

### Single-particle imaging of gaseous I_2_ adsorption

The imaging of gaseous I_2_ adsorption on single ZIF-90 particles is performed using Olympus BX53 optical microscopy setup with a white light source (halogen lamp, 100 W), dark-field condenser (numerical aperture: 1.2–1.4), chip true-color charge-coupled device camera, and 100× immersion objective (numerical aperture: 0.75). The ZIF-90 particles dissolved in ethanol solution are drop-casted onto a glass slide, and dried in air. Then, a borosilicate coverslip and two micropipes are covered on the surface of the glass slide, and sealed by double-sided tape to fabricate a microchamber (2 cm × 1.5 cm × 1.5 mm). Soon after, the gaseous I_2_ with a concentration of 1 mM is successively pumped into the reactor cell, and the excessive gaseous I_2_ is adsorbed by ethanol. The scattering spectra and DFM image are collected by a fiber optic spectrometer (EK2000, Shanghai Choptics).

For imaging the gaseous I_2_ adsorption on single defected ZIF-90 and ZIF-91 particles, the same experimental operations are performed.

### Characterization

The morphological characteristics of the ZIF particles are investigated by using Zeiss Merlin field-emission transmission electron microscope (TEM, Libra200, accelerating voltage: 200 kV) and scanning electron microscope (SEM, UItra55). Elemental distribution diagram is collected by energy dispersive X-ray spectroscopy (EDS) connected with field-emission SEM. The crystal structures are studied by X’ Pert Pro X-ray diffractometer. Specific surface area of the ZIF particles are collected from nitrogen physisorption isotherms at 77 K. The specific surface area and pore size distribution are calculated with Saito-Foley (SF) model^28^ by employing an Autosorb iQ-MP/XR gas sorption analyzer (Quantachrome instruments). The chemical compositions of the samples are investigated by ESCALAB 250Xi X-ray photoelectron spectrometer (XPS), and Shimadzu UV-1900 UV/VIS spectrometer.

## Supplementary information

Final Supplementary Information

Supplementary Movie 1

Supplementary Movie 2

Description of additional supplementary files

## Data Availability

The data that support the findings detailed in this study are available in the Article and its Supplementary Information or from the corresponding authors upon reasonable request. Source data are provided with this paper.

## Source data

Source data
