# Supplementary material for: Visualization of gaseous iodine adsorption on single zeolitic imidazolate framework-90 particles
Source: Nat Commun. 2021 Jul 23;12:4483. doi: 10.1038/s41467-021-24830-1 (PMC8302588; doi:10.1038/s41467-021-24830-1)
Supplement: Supplementary file 1 — Final Supplementary Information [file 41467_2021_24830_MOESM1_ESM.pdf]

Supplementary Information

Visualization of gaseous iodine adsorption on single zeolitic imidazolate framework-90 particles

Yuting Lei[1], Guihua Zhang[1], Qinglan Zhang[1], Ling Yu[1], Hua Li[2], Haili Yu[*1], Yi He[* 1]

[1] National Collaborative Innovation Center for Nuclear Waste and Environmental Safety, School of National Defence Science & Technology, Southwest University of Science and Technology, Mianyang 621010, P. R. China.

[2] SUSTech Core Research Facilities, Southern University of Science and Technology, Shenzhen 518055, China.

*Corresponding author: Dr. Haili Yu and Prof. Dr. Yi He, Tel: +86-816-6089885, Fax: +86-816-6089889, Email: yuhaili-119@163.com, yhe2014@126.com.

Theoretical calculations. The molecular dynamics (MD) simulations were performed with the Forcite code using a supercell consisting of 8 unit cells of ZIF-90 with a cell parameter of 34.54 Å. Framework atoms remained fixed at their crystallographic coordinates throughout the simulations. The energy expression included only short-range van der Waals interactions, with parameters taken from the Universal Force Field (UFF) without modification. A short range cutoff of 17.0 Å was applied. The use of UFF parameters and fixed framework atoms has been used successfully to study the structure and dynamics of adsorbed guests. Guest I_2 molecules were treated with diatomic model, and the van der Waals (vdW) parameters for I atom were taken from the UFF. The simulations were performed in the canonical ensemble with a thermostat temperature of 298 K and a time step of 1.0 fs. Simulations were run for 2.0 ns, with data from the final 1.0 ns used for averaging.

The calculation of the optical absorption spectra was based on the density functional theory. The exchange-correlation potential is described by generalized gradient approximation (GGA) with Perdew-Burke-Ernzerhof (PBE) scheme. Ultrasoft pseudopotentials were generated on the fly. The plane wave approach was used for describing the electron-ion interaction with a cut-off energy of 600 eV. The K-space integrations were conducted using 3 × 3 × 3 Γ-centered k meshes in the first Brillouin-zone. Tkatchenko-Scheffler (TS) scheme was used for vdW dispersion corrections.

Correlation of the adsorption amount of I_2 and B value of the DMF image. In order to correlate the adsorption amount (q) of I_2 per ZIF-90 nanoparticles with the B value change ($\triangle I_B$) of the DMF image, 4 mg ZIF-90 particles are added to 4 mL n-hexane solution with different concentrations of I_2 (0.05, 0.1, 0.3, 0.5, and 0.8 g/L). After incubation at room temperature (25 °C) for 4 h, the reaction solution is centrifuged. The ZIF-90 particles before and after adsorption of I_2 are observed by DMF. We can obtain the averaged $\triangle I_B$ value per ZIF-90 particles via analyzing the DMF images. The residual mass concentration of I_2 in the supernatants (C_r) are calculated based on the standard curve found from the UV/Vis absorbance by using the Beer-Lambert law

(Supplementary Fig. 13). Accordingly, the overall adsorption amount (Q) of I_2 by 4 mg ZIF-90 particles can be estimated by:

$$Q = (C_0 - C_r) * V \qquad \text{(S1)}$$

Where C_0 is the initial mass concentration of I_2, V is the solution volume (V= 4 mL). The average adsorption amount of I_2 per unit mass of ZIF-90 particles (q) is expressed by:

$$q = Q/m \qquad \text{(S2)}$$

We plot $\triangle I_B$ to q in Figure 1f, and a good linear relationship, $\triangle I_B = 0.88q + 17.22$. Because the obtained ZIF-90 particles have rhombic dodecahedron crystals with the most abundant side-length of 1.42 μm (a = 1.42 μm), we can get the volume of single ZIF-90 ($V_{ZIF\text{-}90}$) particle by:

$$V_{ZIF\text{-}90} = \frac{16\sqrt{3}}{9} a^3 \qquad \text{(S3)}$$

Taking the known density of ZIF-90 (ρ=0.974 g/cm^3), the mass of single ZIF-90 particle (m_0) can be calculated by:

$$m_0 = \rho * V_{ZIF\text{-}90} \qquad \text{(S4)}$$

Therefore, the adsorption amount of I_2 for single ZIF-90 particle (q_0) can be calculated by:

$$q_0 = q * m_0 \qquad \text{(S5)}$$

Movies. The uploaded video (movie 1) have compressed three times to reduce its space occupation.

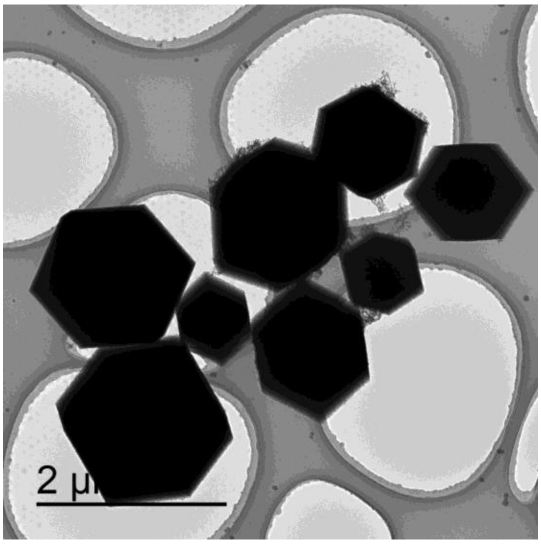

Supplementary Figure 1. TEM image of ZIF-90 particles.

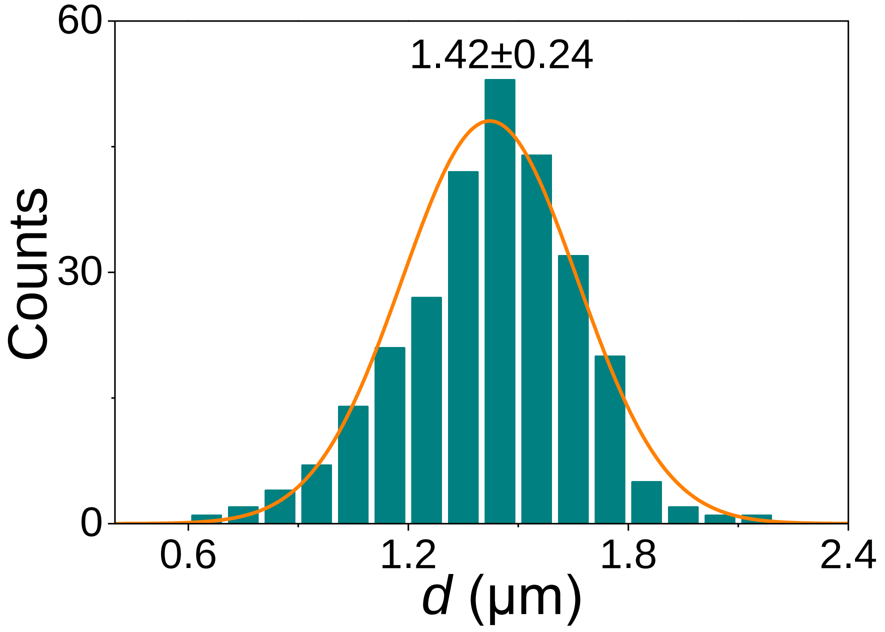

Supplementary Figure 2. Particle size distribution histogram of ZIF-90 particles.

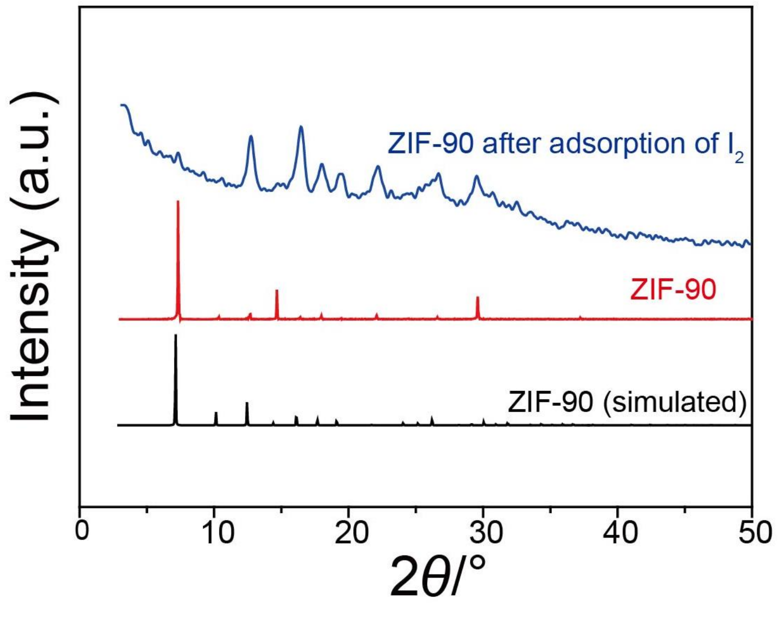

Supplementary Figure 3. XRD patterns of the ZIF-90 before and after adsorption of I_2.

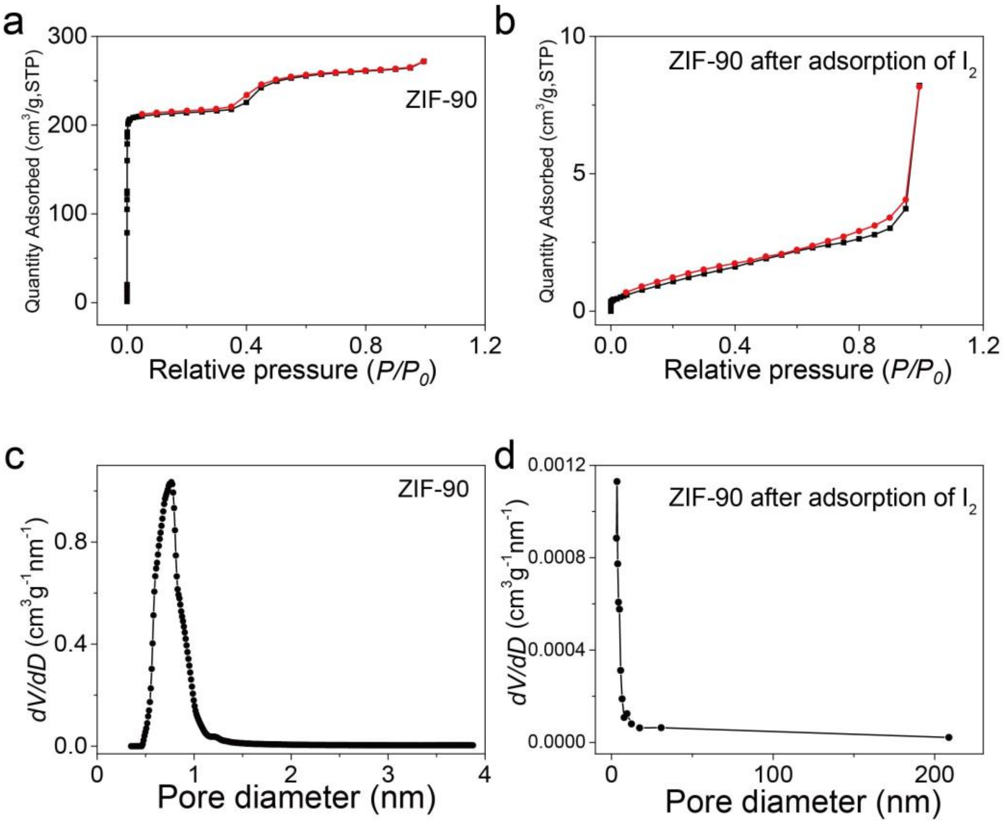

Supplementary Figure 4. (a, b) N_2 adsorption-desorption isotherms and (c, d) pore size distributions of ZIF particles before and after adsorption of I_2.

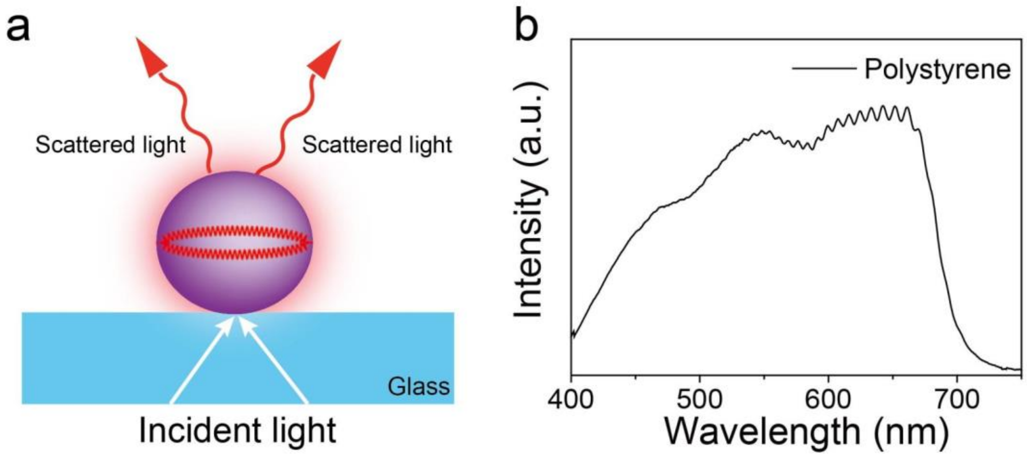

Supplementary Figure 5. (a) Schematic illustration of the light scattering by the WGM resonator. (b) the scattering specrum of polystyrene microspheres with diameter of 10 μm.

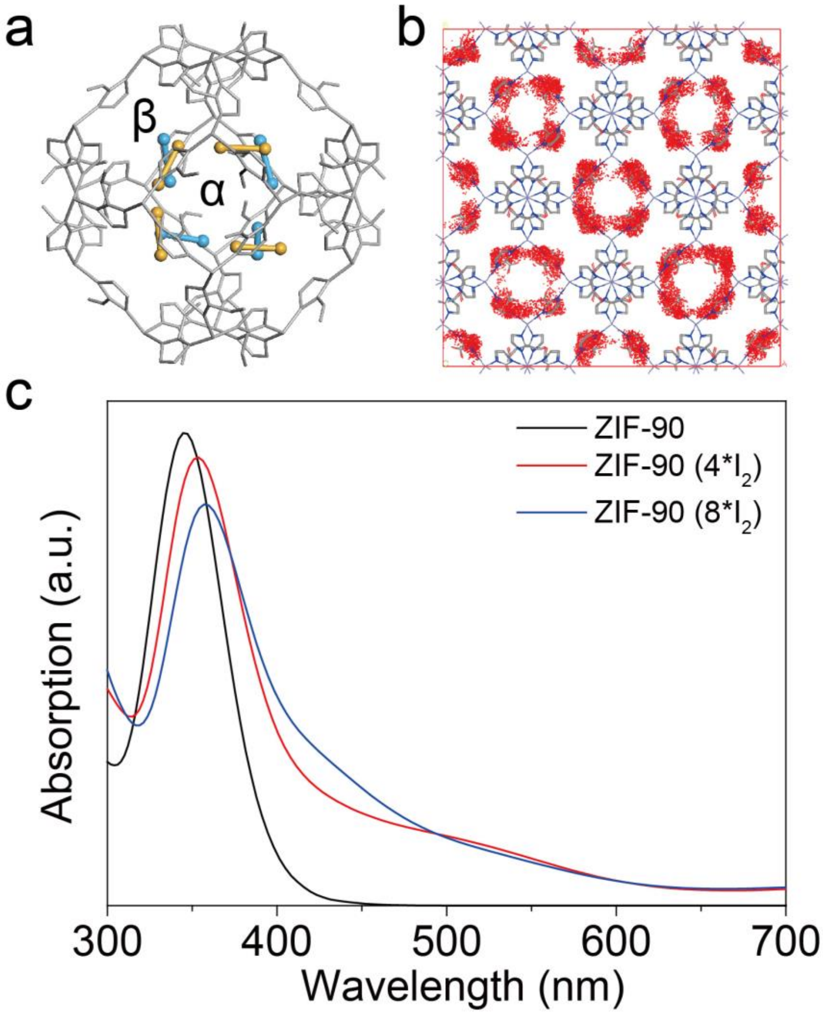

Supplementary Figure 6. (a) Possible adsorption sites of I_2 within ZIF-90 cage. (b) The time-averaged atomic density plot from the MD simulations. (c) The calculated UV-vis absorption spectra of ZIF-90 with different I_2 loading amounts.

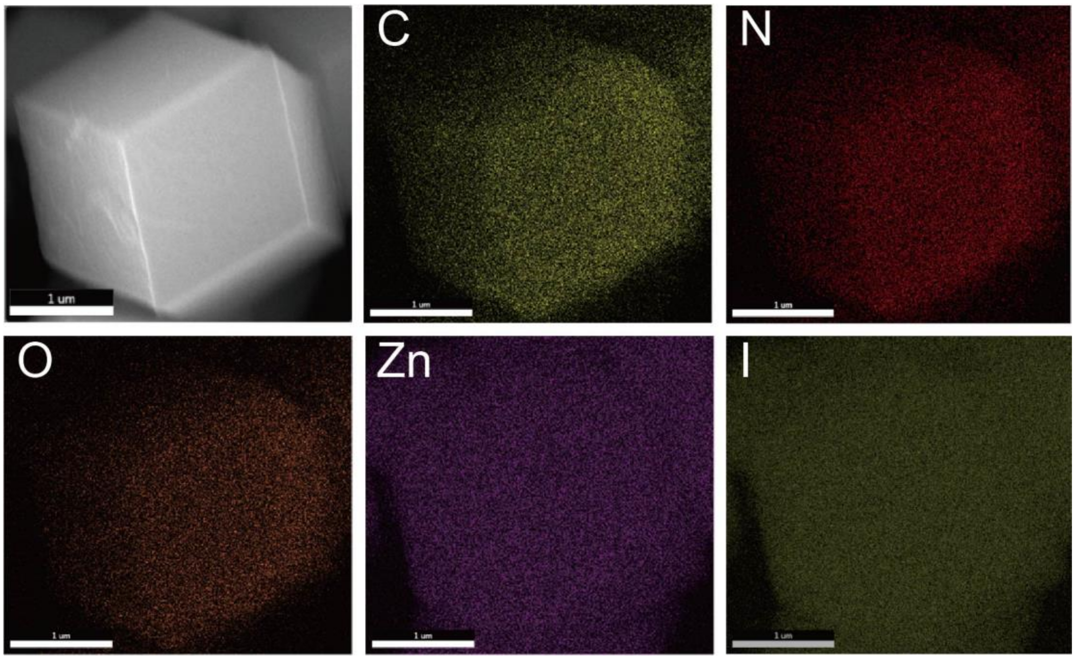

Supplementary Figure 7. SEM image and elemental mapping of ZIF-90 particles after adsorption of I_2 (scale bar: 1 μm).

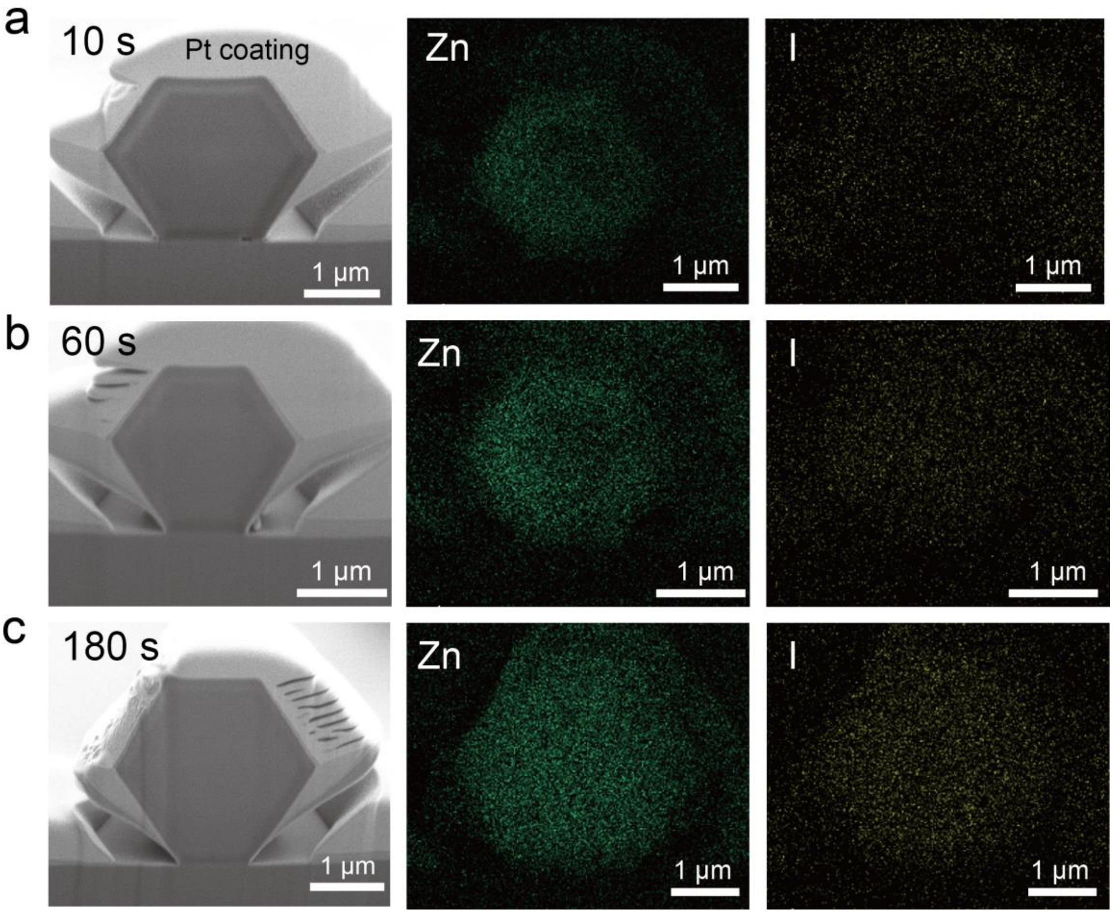

Supplementary Figure 8. SEM images and elemental mapping of I_2-loaded ZIF-90 slices created by FIB under different adsorption time: a) 10 s, b) 60 s, and c) 180 s.

a

	10 s	60 s	180 s
I wt%	9	12.00	13.00
Zn wt%	38	38.00	38.00
I/Zn molar ratio	0.12	0.16	0.18

b

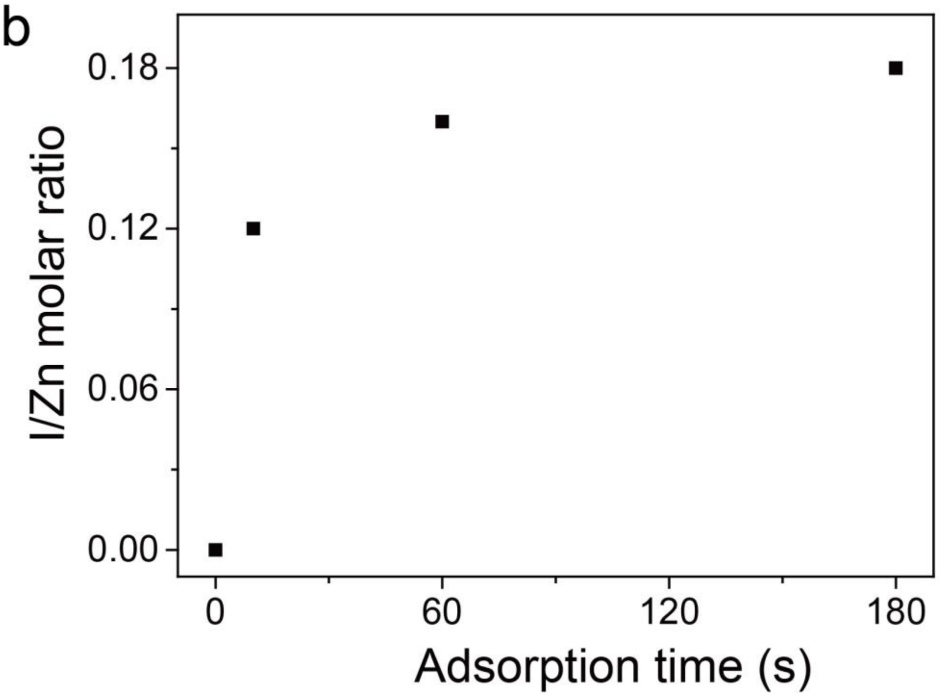

Supplementary Figure 9. (a) Energy-dispersive spectroscopy analysis of Zn and I elements in I_2-loaded ZIF-90 slices under different adsorption time. (b) Plot of the I/Zn molar ratio as a function of adsorption time.

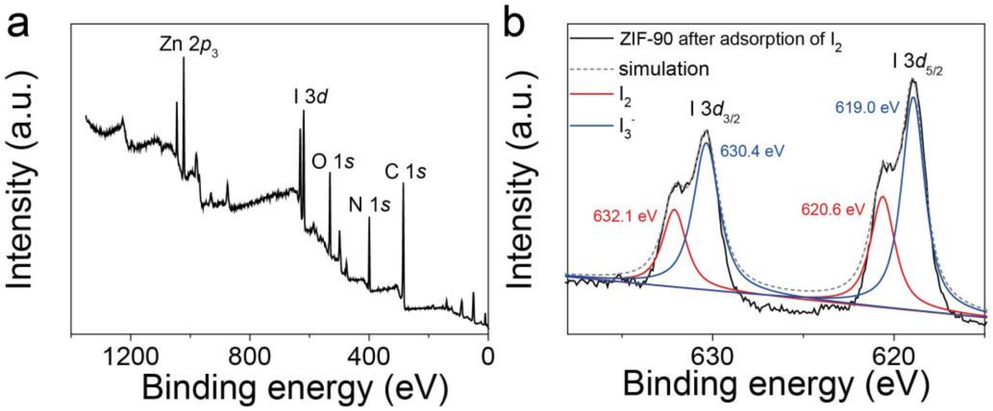

Supplementary Figure 10. (a) XPS survey scan and (b) I 3*d* peaks of ZIF-90 particles after adsorption of I_2.

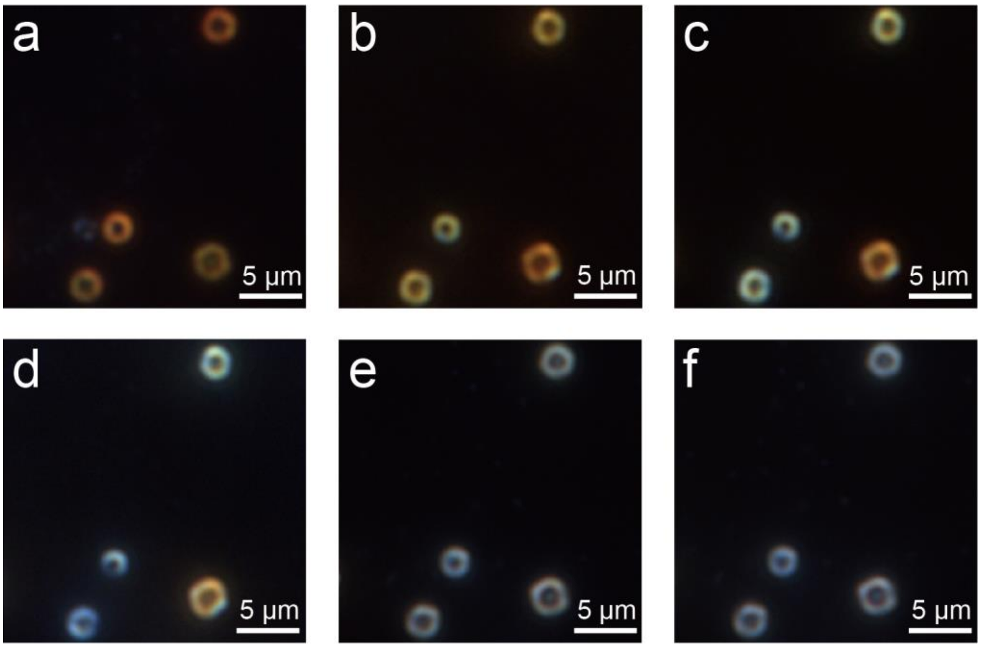

Supplementary Figure 11. DFM images of I_2-loaded ZIF-90 particles (a) before and after washing different times ((b) once, (c) twice, (d) three times, (e) four times and (f) five times) with absolute EtOH.

As shown in Supplementary Fig. 11, the yellow I_2-loaded ZIF-90 particles gradually turns to bluewhite after washing different times with absolute EtOH, confirming that the adsorbed I_2 can be eluted from ZIF-90 particles and this adsorption process is reversible.

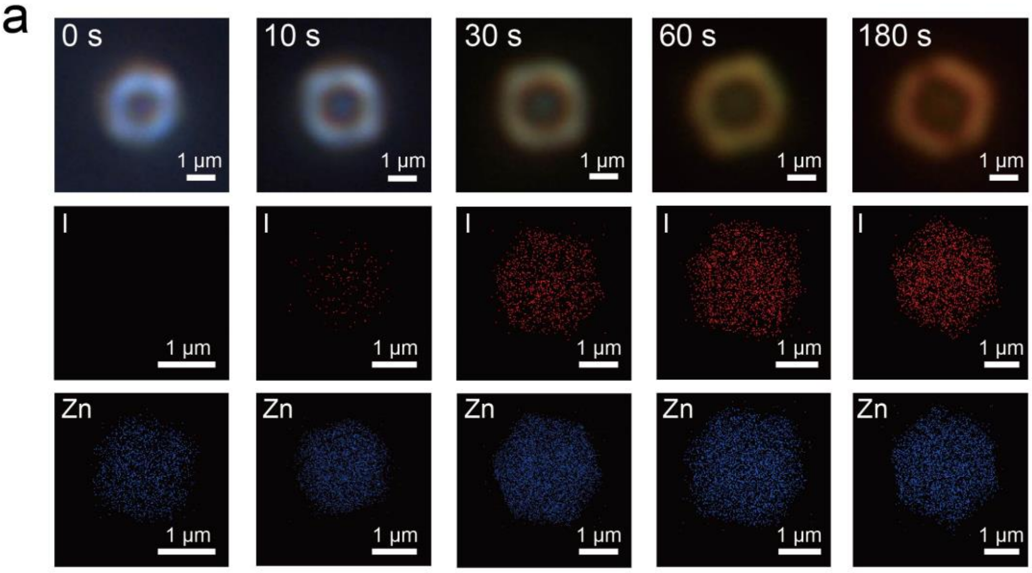

b

	0 s	10 s	30 s	60 s	180 s
I wt%	0	2.58	30.36	37.49	36.64
Zn wt%	91.25	74.43	62.42	33.83	26.78
I/Zn molar ratio	0	0.02	0.25	0.57	0.70

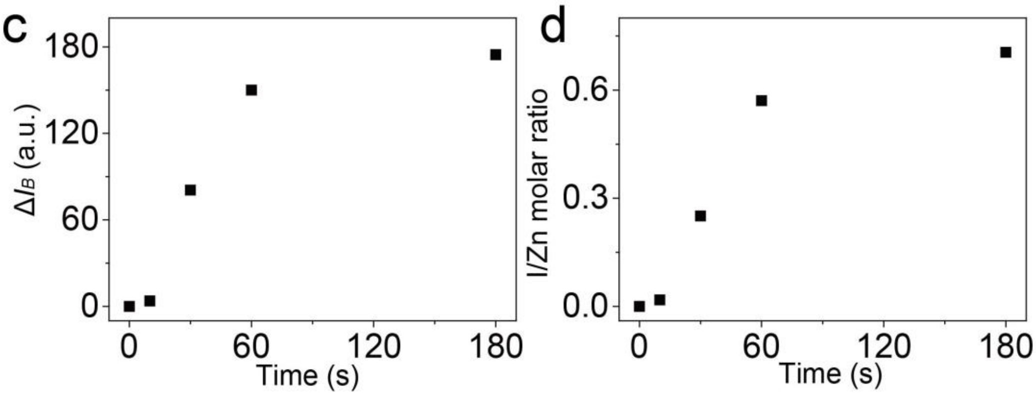

Supplementary Figure 12. (a) Time-lapsed DFM images of gaseous I_2 adsorption on single ZIF-90 particles and the corresponding TEM-EDS mapping images. (b) The contents of I and Zn elements in single ZIF-90 particles under different adsorption time. Plots of (c) the change of B value ($\triangle I_B$) from the DFM image and (d) I/Zn molar ratio versus adsorption time.

The DFM images and the corresponding X ray/synchrotron data are shown in Supplementary Fig. 12. It can be seen that the color of the ZIF-90 particle under DFM

observation becomes yellow gradually, accompanying the continuous increase of the I/Zn molar ratio (Supplementary Fig. 12a, 12b and 12d). More importantly, both the change of B value ($\triangle I_B$) from the DFM image and I/Zn molar ratio follow a similar trend with increasing absorption time (Supplementary Fig. 12c and 12d), revealing that the color change of the DFM image is induced by adsorption of gaseous I_2 on ZIF-90 particles.

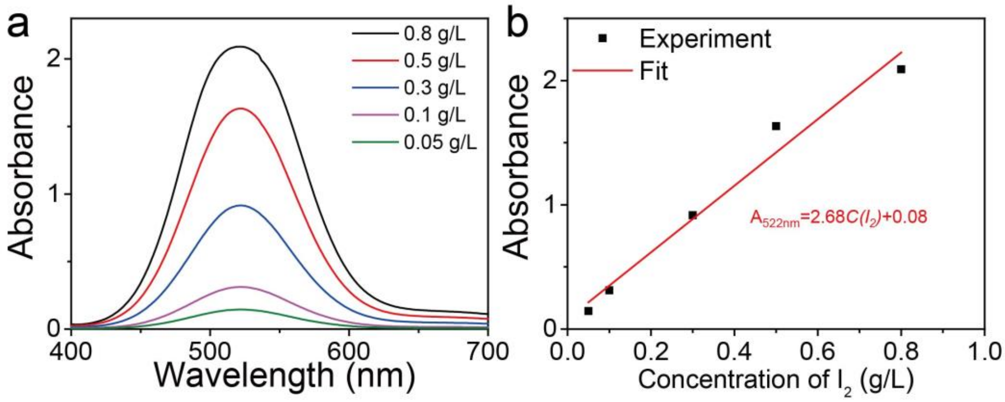

Supplementary Figure 13. (a) UV-vis absorption spectra of I_2 with different concentrations (0.05 g/L-0.8 g/L) in hexane solution. (b) The calibration plot for I_2 concentration vs absorbance.

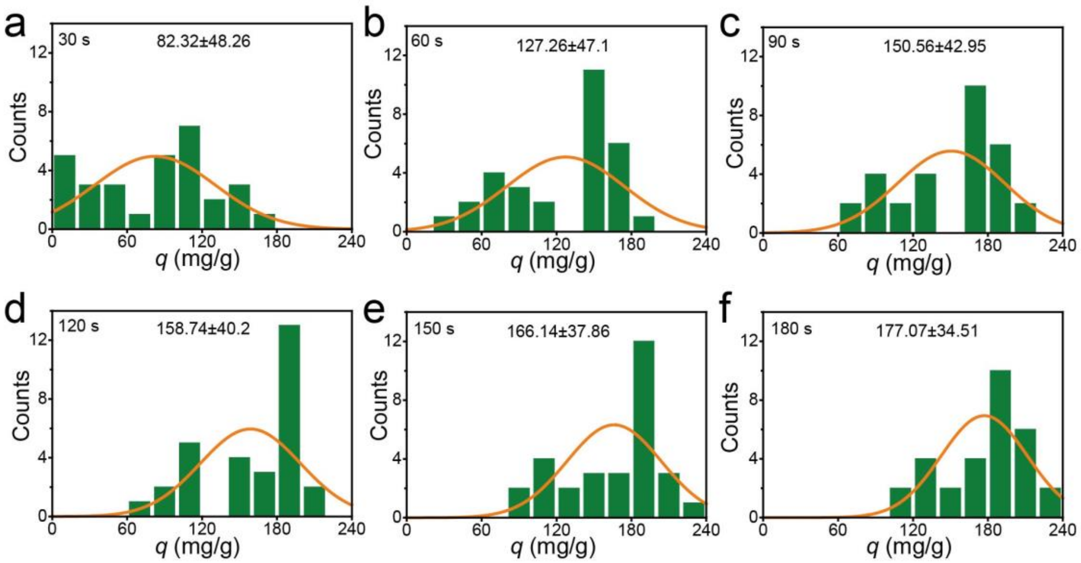

Supplementary Figure 14. Statistical distribution of the adsorption amount for single ZIF-90 particles at different reaction time.

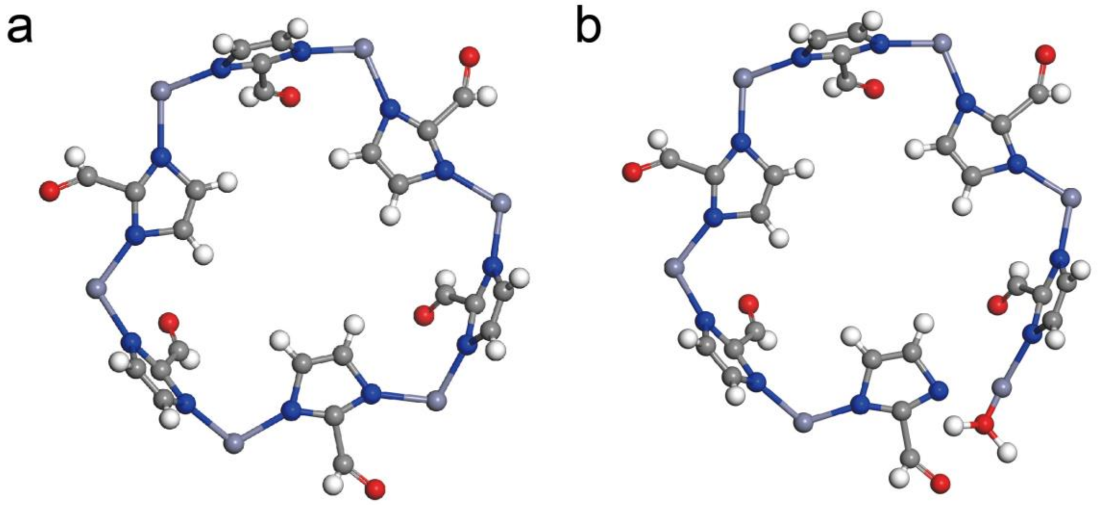

Supplementary Figure 15. Local structures of ZIF-90 in the (a) absence and (b) presence of the linker defect (dangling linker).

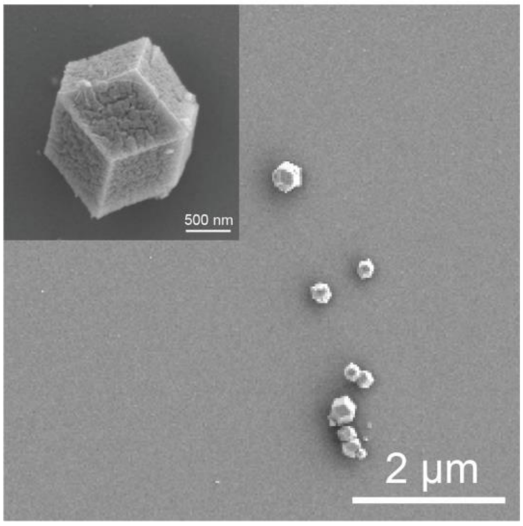

Supplementary Figure 16. SEM image of defected ZIF-90 particles.

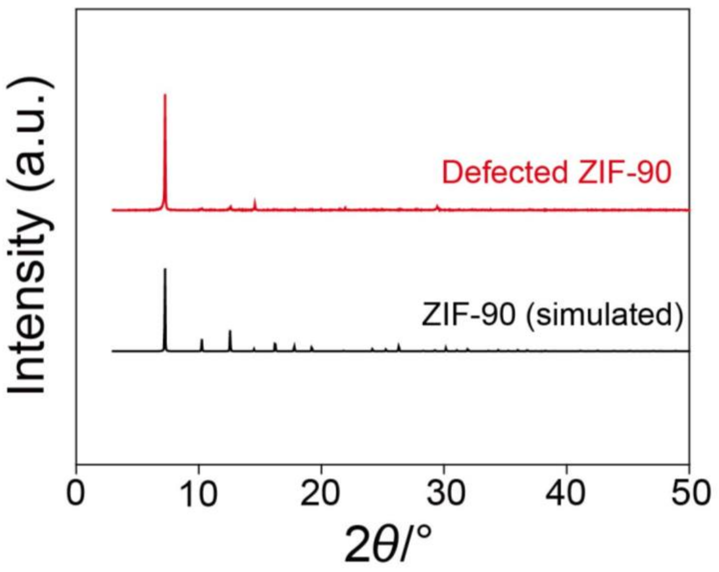

Supplementary Figure 17. XRD pattern of the resulting defected ZIF-90 particles.

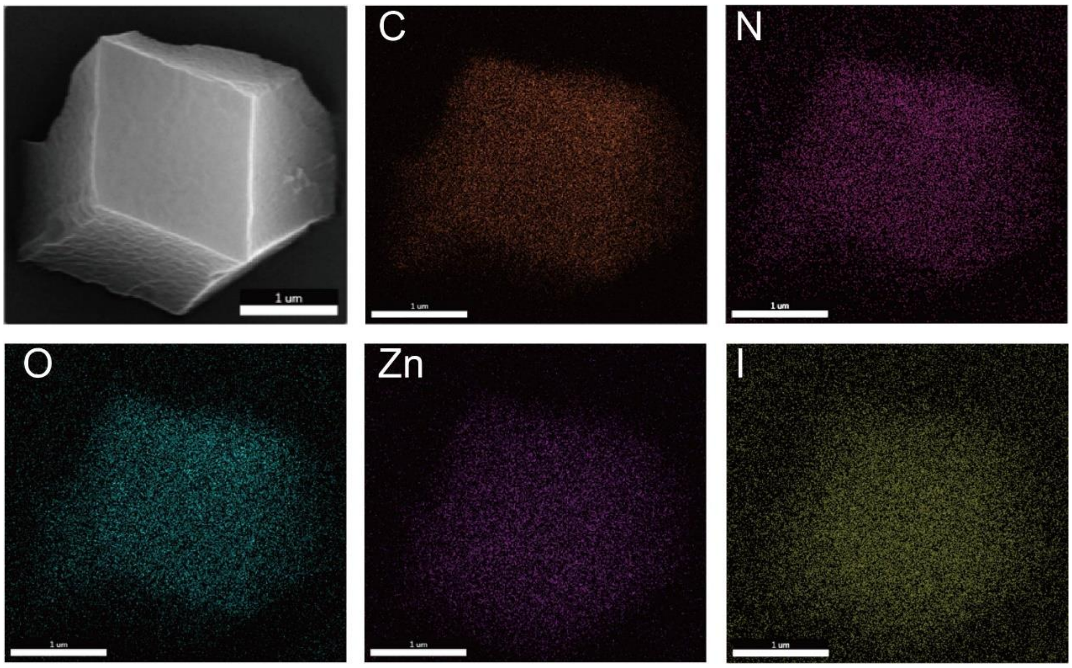

Supplementary Figure 18. SEM image and elemental mapping of defected ZIF-90 particles after adsorption of I_2.

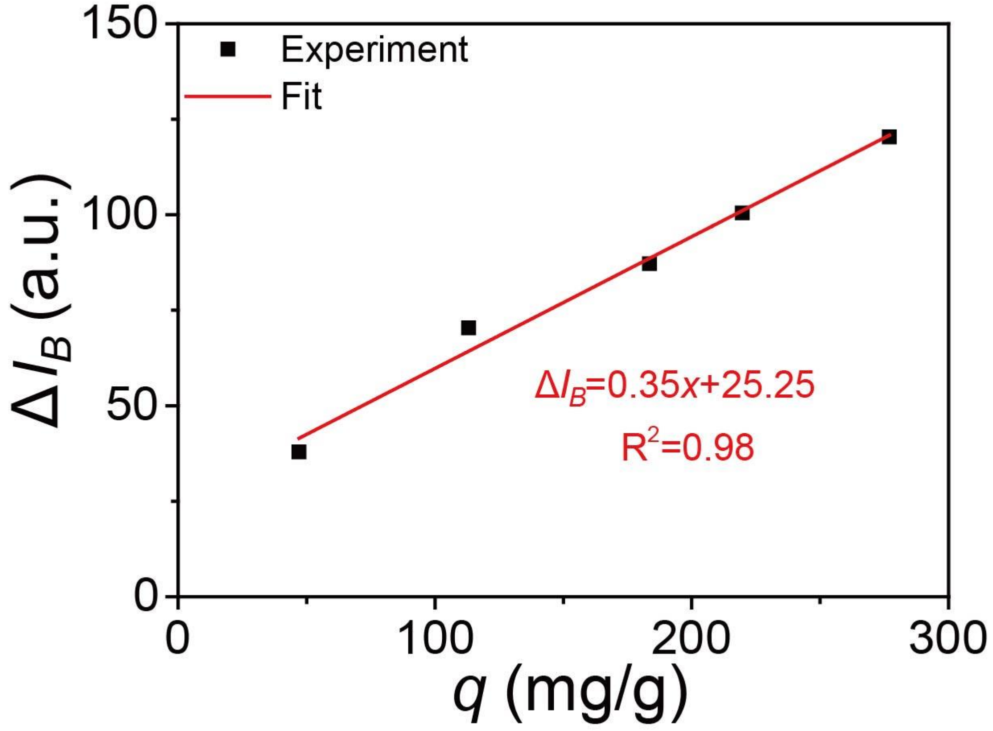

Supplementary Figure 19. Plot of the change of B value ($\triangle I_B$) from the DFM image as a function of adsorption amount of I_2 (q) by defected ZIF-90 particles.

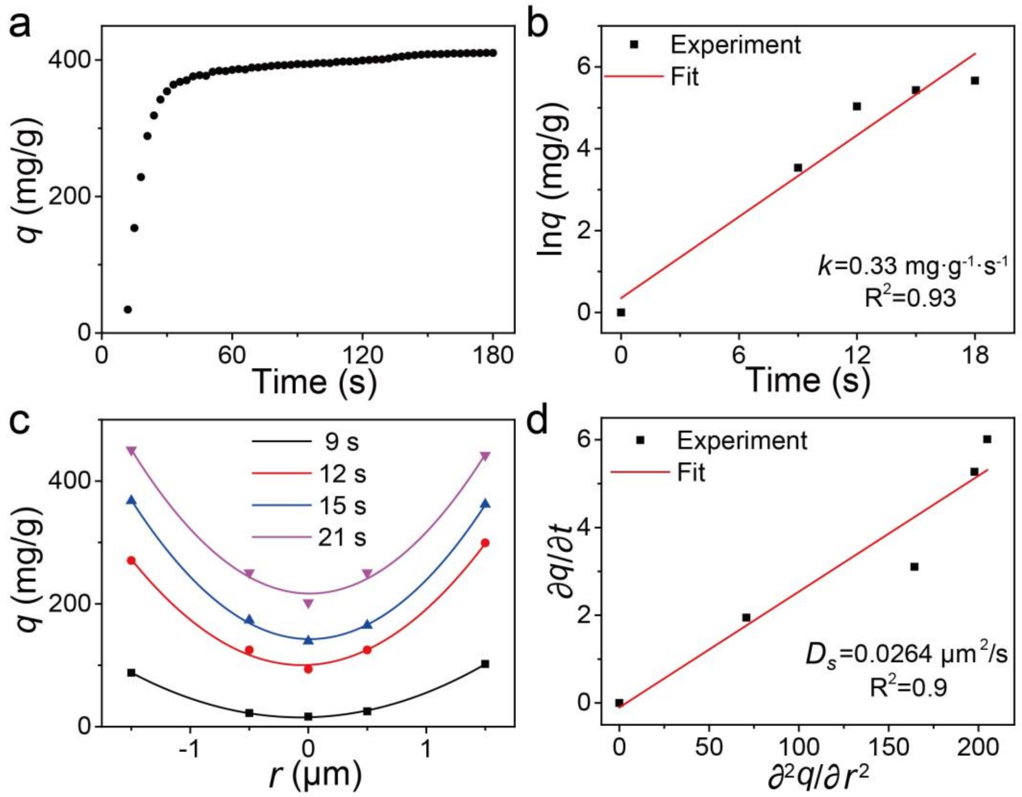

Supplementary Figure 20. (a) Adsorption kinetic of gaseous I_2 onto a single defected ZIF-90 particle. (b) Pseudo-first order model for adsorption of gaseous I_2 on defected ZIF-90 particle. (c) Spatial and temporal variability of I_2 absorption amount distributions in line profiles on single defected ZIF-90 particle. (d) Diffusion coefficient within defected ZIF-90 particle estimated from Fick's second law.

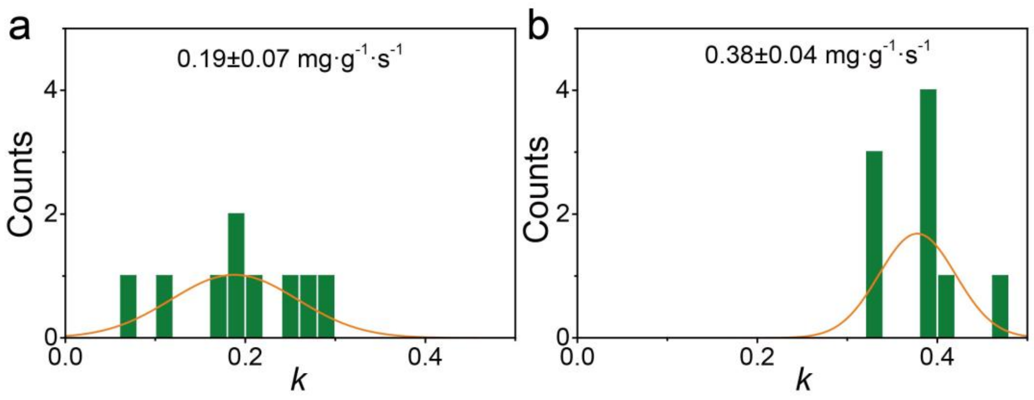

Supplementary Figure 21. Distribution of the adsorption rate constants for ZIF-90 and defected ZIF-90 particles.

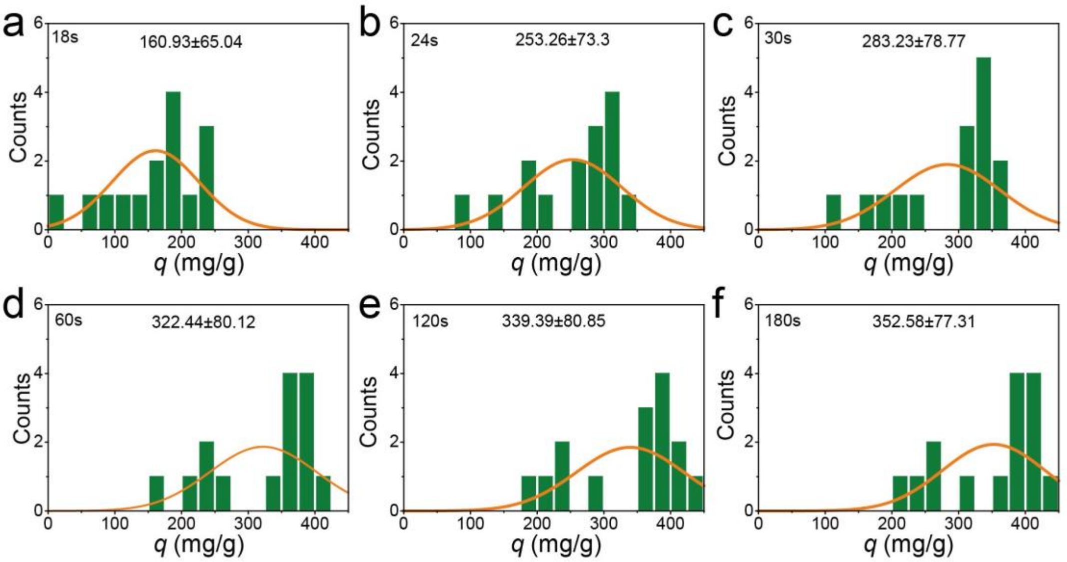

Supplementary Figure 22. Distribution of the adsorption amount for defected ZIF-90 particles at different reaction time.

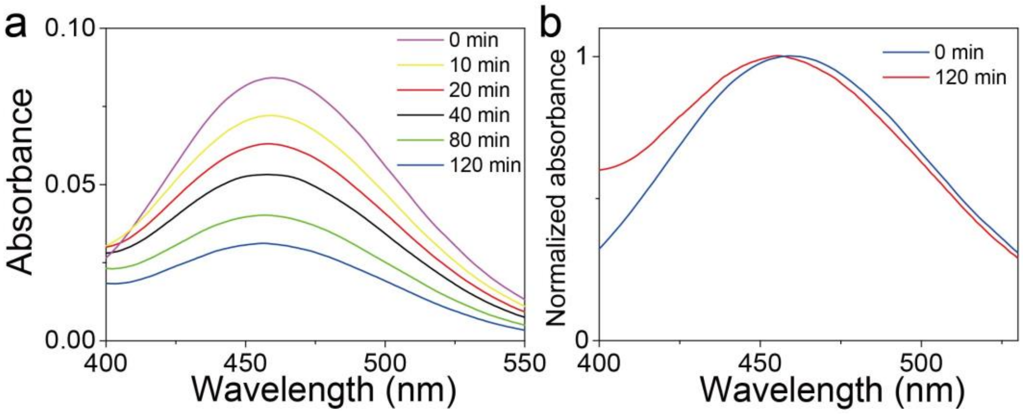

Supplementary Figure 23. (a) Time-dependent UV-vis absorption spectra of I_2-imidazole-2-carboxaldehyde aqueous solution. (b) Normalized absorption spectra of I_2-imidazole-2-carboxaldehyde before and after reaction for 120 min. The mass concentrations of I_2 and imidazole-2-carboxaldehyde are 0.1 g/L.

As shown in Supplementary Fig. 23, the absorption band of I_2 at 460 nm in aqueous solution is blueshifted to 454 nm, indicating the generation of I_2-imidazole-2-carboxaldehyde CT complex[1].

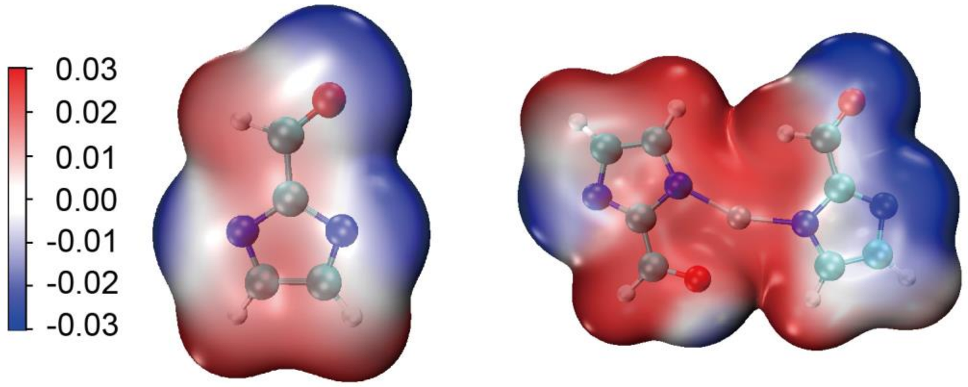

Supplementary Figure 24. Electrostatic potential (ESP) maps of imidazole-2-carboxaldehyde before and after coordination with Zn^{2+}.

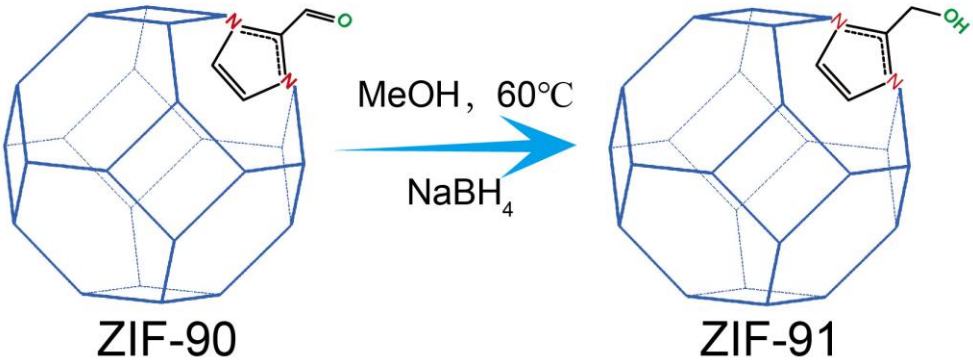

Supplementary Figure 25. Schematic illustration of preparation of ZIF-91 particles by reduction of ZIF-90.

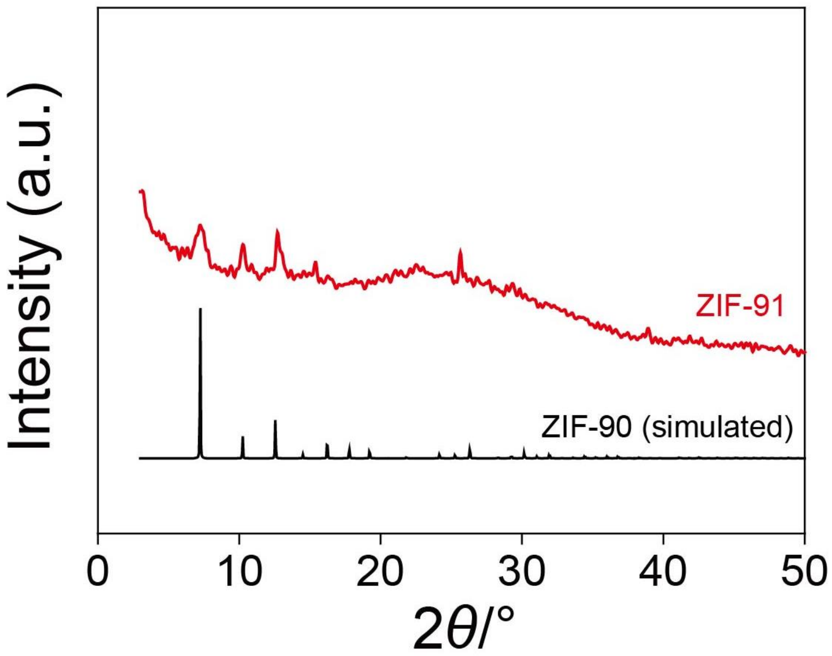

Supplementary Figure 26. XRD pattern of the resulting ZIF-91 particles.

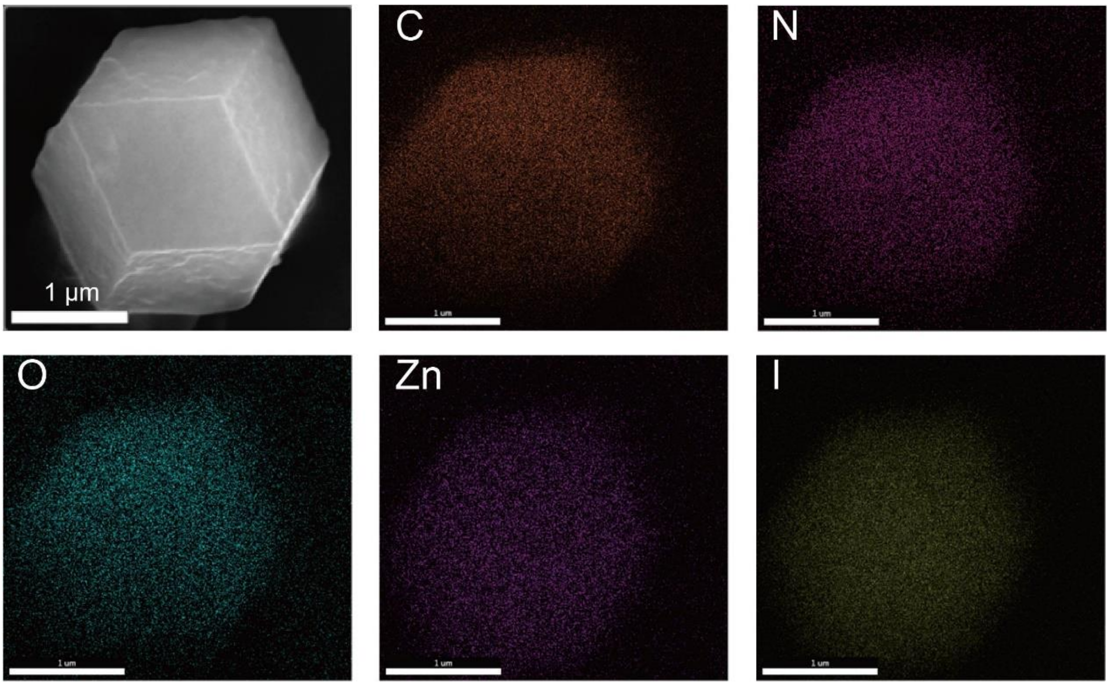

Supplementary Figure 27. SEM image and elemental mapping of the ZIF-91 particle after adsorption of I_2.

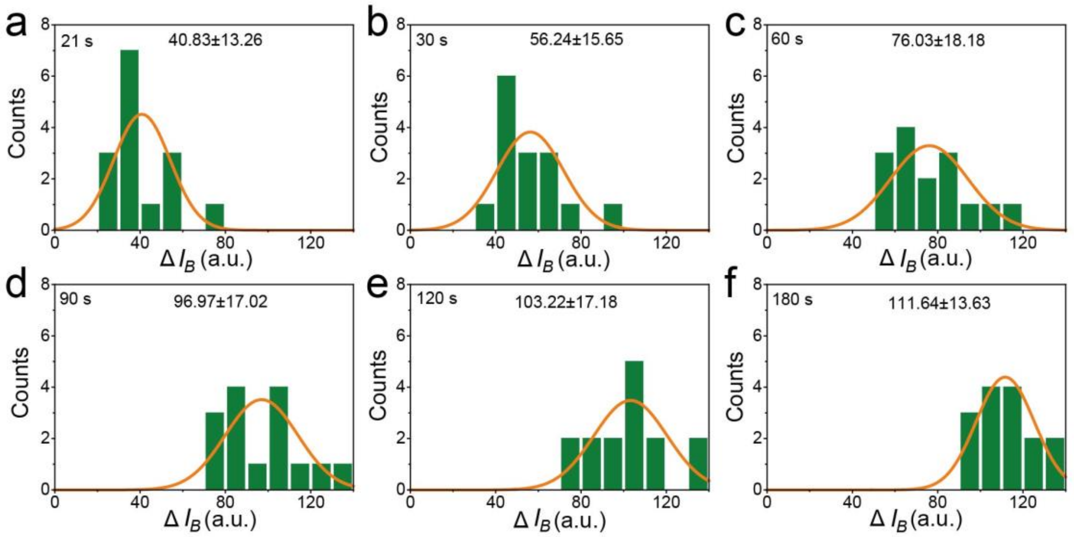

Supplementary Figure 28. Statistical distribution of the adsorption amount for ZIF-91 particles at different reaction time.

Supplementary references

1 Nagakura, S. Molecular Complexes and their Spectra. VIII. The Molecular Complex between Iodine and Triethylamine. *J. Am. Chem. Soc.* **80**, 520-524 (1958).
