# Supplementary material for: Visualization of gaseous iodine adsorption on single zeolitic imidazolate framework-90 particles
Source: Nat Commun. 2021 Jul 23;12:4483. doi: 10.1038/s41467-021-24830-1 (PMC8302588; doi:10.1038/s41467-021-24830-1)
Supplement: Supplementary file 4 — Description of additional supplementary files [file 41467_2021_24830_MOESM4_ESM.docx]

Description of additional supplementary information

Title: Supplementary Movie 1

Description: DFM imaging of gaseous iodine adsorption on single ZIF-90 particle

Title: Supplementary Movie 2

Description: Molecular dynamics simulation of the diffusion of gaseous iodine within ZIF-90
